# Innovative Micro- and Nano-Architectures in Biomedical Engineering for Therapeutic and Diagnostic Applications

**DOI:** 10.3390/mi16040419

**Published:** 2025-03-31

**Authors:** Nargish Parvin, Sang Woo Joo, Jae Hak Jung, Tapas K. Mandal

**Affiliations:** 1 School of Mechanical Engineering, Yeungnam University, Gyeongsan 38541, Republic of Korea; nargish.parvin@gmil.com (N.P.); swjoo@yu.ac.kr (S.W.J.); 2 School of Chemical Engineering, Yeungnam University, Gyeongsan 38541, Republic of Korea

**Keywords:** micro- and nano-engineered biomaterials, biomedical microstructures, advanced drug delivery systems, nanotechnology in diagnostics, microfabrication for healthcare

## Abstract

The rapid evolution of micro- and nano-architectures is revolutionizing biomedical engineering, particularly in the fields of therapeutic and diagnostic micromechanics. This review explores the recent innovations in micro- and nanostructured materials and their transformative impact on healthcare applications, ranging from drug delivery and tissue engineering to biosensing and diagnostics. Key advances in fabrication techniques, such as lithography, 3D printing, and self-assembly, have enabled unprecedented control over material properties and functionalities at microscopic scales. These engineered architectures offer enhanced precision in targeting and controlled release in drug delivery, foster cellular interactions in tissue engineering, and improve sensitivity and specificity in diagnostic devices. We examine critical design parameters, including biocompatibility, mechanical resilience, and scalability, which influence their clinical efficacy and long-term stability. This review also highlights the translational potential and current limitations in bringing these materials from the laboratory research to practical applications. By providing a comprehensive overview of the current trends, challenges, and future perspectives, this article aims to inform and inspire further development in micro- and nano-architectures that hold promise for advancing personalized and precision medicine.

## 1. Introduction

### 1.1. Background on Micro- and Nano-Architectures in Biomedical Engineering

The integration of micro- and nano-architectures within biomedical engineering has significantly advanced over recent decades, driven by the demand for enhanced therapeutic and diagnostic modalities. Micro- and nanostructures are designed to manipulate biological systems at cellular, molecular, and sub-molecular levels, enabling precise control over physiological and pathological processes. These architectures often mimic the hierarchical organization observed in natural systems, such as the extracellular matrix (ECM) and organelles, which offer essential cues for cell behavior and tissue homeostasis [1]. Micro-architectures, typically in the range of 1–1000 μm, have demonstrated profound applications in tissue scaffolding, where their geometric precision facilitates the development of biomimetic environments [2]. In parallel, nano-architectures (<100 nm) exploit quantum and surface effects, allowing enhanced interaction with biomolecules, such as proteins, DNA, and cell membranes [3]. For instance, nanostructures can improve drug bioavailability by crossing biological barriers like the blood–brain barrier [4,5]. The convergence of multidisciplinary fields, including material science, nanotechnology, and bioengineering, has played a pivotal role in driving these advancements. By leveraging innovative fabrication tools, such as lithography and self-assembly, researchers have unlocked new paradigms in biointerfacing and clinical applications [6]. These technologies not only improve the performance of existing devices, but also pave the way for novel solutions to complex medical challenges. Despite these advances, several challenges remain in translating micro- and nano-architectures into widespread clinical use. The current limitations include the lack of precise control over biomaterial interactions at the molecular level, difficulties in large-scale manufacturing, and concerns regarding long-term stability and biocompatibility. In drug delivery, achieving targeted therapeutic release without off-target effects remains a critical hurdle, while in tissue engineering, the ability to replicate the dynamic and heterogeneous nature of native tissues is still under development. Similarly, diagnostic microfluidic platforms face scalability challenges, limiting their integration into routine clinical workflows. This review aims to address these gaps by critically evaluating recent breakthroughs that enhance specificity, scalability, and functional longevity in biomedical applications.

Micro- and nano-architectures can be designed using a diverse range of materials, including polymers, inorganic compounds, natural biomaterials, and even robotic systems. These materials are selected based on their intended applications, such as biocompatibility, mechanical properties, and functionality at the micro- and nanoscale. Polymeric materials, such as polylactic acid (PLA), polyethylene glycol (PEG), and polycaprolactone (PCL), are frequently employed due to their versatility, biocompatibility, and ability to be easily processed into complex micro- and nanostructures for drug delivery and tissue scaffolding [7]. Inorganic materials, such as silica, gold, and iron oxide nanoparticles, offer distinct advantages in terms of stability and optical or magnetic properties, making them ideal for imaging and contrast-enhancing applications [8]. Natural biomaterials, like chitosan, collagen, and alginate, are increasingly being used for their ability to promote cell adhesion, differentiation, and tissue regeneration [9]. Robotic systems and bioinspired techniques, such as self-assembly and 3D printing, enable the creation of highly functional micro- and nano-architectures with precise control over shape, size, and material composition [10,11]. These techniques allow for the fabrication of structures with tailored properties, whether for enhancing cellular interactions in tissue engineering or for developing efficient drug delivery systems. The materials used in micro- and nano-architectures for biomedical applications are presented in Table 1.

### 1.2. Importance in Healthcare Applications

Micro- and nano-architectures are at the forefront of revolutionizing healthcare by addressing the unmet clinical needs. In drug delivery, these architectures enable the development of carrier systems that provide sustained and targeted release of therapeutics, reducing off-target toxicity while maximizing efficacy [12]. For example, nanoparticle-based drug carriers functionalized with ligands have shown exceptional promise in tumor-targeted therapies [13,14,15]. Additionally, nanostructures like liposomes and dendrimers have already been translated into clinical applications, with products such as Doxil^®^ exemplifying the success of nano-drug delivery systems [16]. In tissue engineering, micro- and nanoscale scaffolds are indispensable for creating artificial tissues and organs [17]. These scaffolds provide structural support while guiding cellular organization and differentiation, closely replicating native tissue functionality. The use of nano-architectures in bone regeneration, for instance, has demonstrated improved osteointegration and mineralization, significantly enhancing the clinical outcomes [18,19]. Furthermore, advancements in microfluidic devices and lab-on-a-chip platforms have enabled the miniaturization of diagnostic tools, allowing the rapid, cost-effective, and accurate detection of biomarkers for diseases like cancer and infectious disorders [20,21]. Many current medical technologies face critical limitations, including inefficient drug targeting, slow tissue regeneration, and inadequate real-time disease monitoring. Traditional therapeutic strategies often lack precision, leading to systemic toxicity and reduced treatment efficacy, while conventional diagnostics rely on bulky, labor-intensive procedures with delayed results. By leveraging micro- and nano-architectures, the researchers aim to bridge these gaps, offering highly specific, scalable, and patient-personalized solutions for improved clinical outcomes. The importance of these architectures is further underscored by their role in advancing personalized medicine. By integrating patient-specific data, micro- and nano-devices can be tailored to meet individual therapeutic and diagnostic requirements. This approach is particularly valuable in managing chronic conditions such as diabetes, where smart drug delivery systems provide real-time glucose monitoring and insulin release.

### 1.3. Scope and Objectives of This Review

This review aims to provide a comprehensive analysis of the recent innovations in micro- and nano-architectures, focusing on their transformative impact on therapeutic and diagnostic micromechanics. The scope encompasses a detailed examination of advanced fabrication techniques, including lithography, 3D printing, and self-assembly, which have enabled precise control over material properties and functionalities [22]. Their applications in drug delivery, tissue engineering, and diagnostic platforms, highlight their ability to address complex clinical challenges through enhanced precision and performance [23]. A critical evaluation of design parameters, such as biocompatibility, mechanical resilience, and scalability, forms the backbone of this review. These factors are essential for translating micro- and nano-architectures from the laboratory to clinical settings, where long-term stability and regulatory compliance are key considerations. By exploring the emerging trends and identifying gaps in the current research, this article aims to inspire new directions in the field, aligning with the goals of personalized and precision medicine [24]. Additionally, this review acknowledges the challenges and limitations associated with these technologies, such as issues of scalability, cost-effectiveness, and ethical considerations in their application. By addressing these aspects, we provide a roadmap for future research that seeks to overcome these barriers and maximize the clinical utility of micro- and nano-architectures. Ultimately, this review aspires to serve as a resource for researchers, clinicians, and industry professionals working at the interface of material science and biomedical engineering. By bridging the gap between foundational research and translational applications, we aim to contribute to the ongoing evolution of micro- and nanotechnologies in healthcare.

## 2. Advances in Fabrication Techniques

### 2.1. Lithography-Based Methods

Lithography has emerged as a cornerstone in the fabrication of micro- and nano-architectures, enabling precise control over patterning materials at microscopic and nanoscopic scales. The term lithography refers to a suite of techniques used to transfer geometric patterns onto a substrate, offering exceptional spatial resolution and repeatability [25]. It is widely used in biomedical engineering to create structures that mimic cellular and extracellular environments, manufacture biosensors, and fabricate microfluidic devices [26]. Modern lithographic methods can achieve features with sub-10 nm precision, providing unparalleled opportunities for the development of advanced biomedical devices. Photolithography and electron beam lithography (EBL) are among the most prominent lithography-based techniques, each tailored for specific applications based on their resolution, scalability, and cost-effectiveness.

#### 2.1.1. Photolithography

Photolithography, also known as optical lithography, is one of the oldest and most extensively used lithographic techniques. It relies on ultraviolet (UV) light to transfer patterns from a mask onto a photosensitive polymer known as a photoresist [27]. The patterned photoresist acts as a template for subsequent material deposition or etching processes, allowing the fabrication of intricate micro-architectures. The photolithography process begins with coating the substrate, typically silicon, glass, or polymer, with a thin layer of photoresist. A photomask containing the desired pattern is aligned with the substrate, and UV light is directed through the mask onto the photoresist. Depending on the type of photoresist—positive or negative—the exposed regions either become soluble and are washed away (positive photoresist) or insoluble (negative photoresist) [28]. This step is followed by etching or deposition, which translates the pattern onto the substrate. Photolithography has been instrumental in fabricating microfluidic devices for lab-on-a-chip platforms. For example, the creation of microchannels with precise dimensions allows for the controlled manipulation of biological fluids, enabling applications such as organ-on-a-chip systems and single-cell analysis [29]. Additionally, photolithography has been used to produce bioactive scaffolds for tissue engineering. By integrating photomasks with cellular adhesive regions, the researchers can guide cell attachment and proliferation, mimicking the native tissue microenvironment. Photolithography offers several advantages, including high throughput, scalability, and the ability to create features as small as 100 nm using deep UV light. However, it has limitations, particularly in three-dimensional (3D) patterning. The technique is inherently two-dimensional, requiring additional steps for multilayered or 3D structures [30]. Moreover, the need for cleanroom facilities and expensive photomasks can be prohibitive for small-scale or exploratory research. Emerging trends in photolithography aim to overcome these limitations through innovations, such as interference lithography and nanoimprint lithography, which promise higher resolutions and more efficient processes [31]. Combining photolithography with other techniques, such as 3D printing, is also gaining traction to fabricate hybrid architectures for advanced biomedical applications.

#### 2.1.2. Electron Beam Lithography

Electron beam lithography (EBL) is a highly versatile lithographic technique that utilizes a focused electron beam to write patterns directly onto an electron-sensitive resist. Unlike photolithography, which relies on masks, EBL is maskless, offering unmatched resolution and flexibility [32]. It is particularly suited for applications that require nanoscale precision, such as the fabrication of biosensors and nanostructured surfaces for cellular studies. The EBL process begins with coating a substrate with an electron-sensitive resist, such as polymethyl methacrylate (PMMA). A focused electron beam is then scanned over the resist in a predefined pattern, altering the solubility of the exposed regions. Subsequent development and etching processes transfer the pattern onto the substrate [33]. The use of an electron beam instead of UV light allows for feature sizes as small as 2 nm, far surpassing the resolution of photolithography. EBL has found significant applications in the creation of nanostructured surfaces that regulate cellular behavior. For instance, nanopatterned surfaces fabricated via EBL can direct stem cell differentiation by mimicking the nanoscale topography of the extracellular matrix [34]. Additionally, EBL has been employed to produce nanoarrays for high-throughput drug screening and biomarker detection. In diagnostics, EBL enables the development of plasmonic nanostructures and nanoantennas that enhance the sensitivity of biosensors [35]. These nanostructures amplify the signals from biomolecular interactions, facilitating the detection of diseases at their early stages. The precision of EBL is also critical in fabricating nanofluidic devices, which are used to study single-molecule dynamics and DNA sequencing.

**Figure 1:**
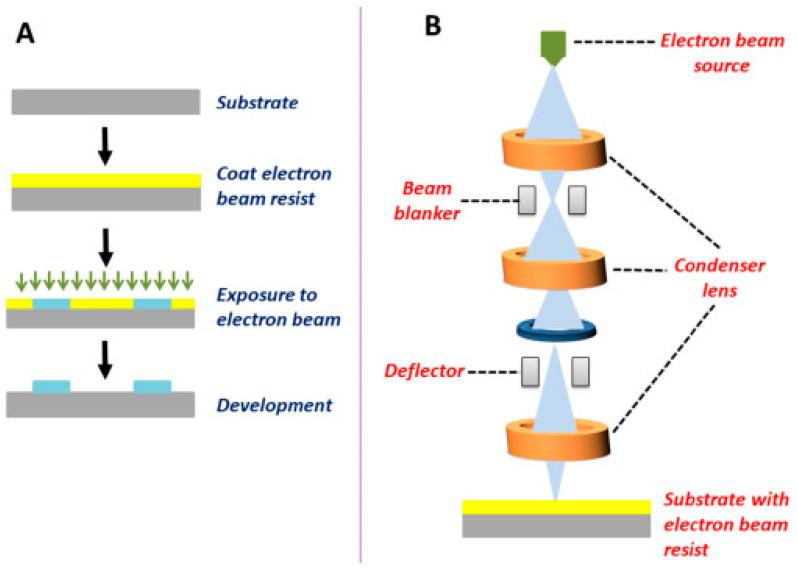
(**A**) Step-by-step process of electron beam lithography fabrication and (**B**) schematic illustration of the operational mechanism of an electron beam lithography system. Reprinted with permission from Ref. [37]. Copyright 2024 Nature Portfolio, Springer Nature.

The primary advantage of EBL is its superior resolution, which allows for the creation of nanostructures that are difficult or impossible to achieve with other techniques. Its maskless nature provides flexibility, making it ideal for prototyping and exploratory research. However, EBL is inherently slow due to the serial writing process, making it unsuitable for high-throughput applications [26]. Additionally, the equipment and operational costs are significantly higher compared to photolithography. Efforts to improve the throughput of EBL focus on parallelizing the writing process using multiple electron beams or integrating it with other fabrication techniques. The researchers are also exploring its potential for hybrid fabrication, such as combining EBL with self-assembly to create complex hierarchical structures [36]. The development of novel resists and advanced electron sources further promises to enhance the capabilities and efficiency of EBL. Figure 1 illustrates the fundamental aspects of electron beam lithography (EBL), a high-resolution patterning technique widely used in micro- and nanofabrication [37]. Figure 1A outlines the sequential fabrication steps involved in EBL, beginning with substrate preparation, followed by the deposition of an electron-sensitive resist layer, electron beam exposure to define nanoscale patterns, and subsequent development to remove the exposed or unexposed regions based on the resist type. The process is completed with metal deposition and lift-off, resulting in precise micro- and nanostructures. Figure 1B presents a schematic representation of the working principle of an EBL system, where a highly focused electron beam is directed onto the resist-coated substrate under vacuum conditions. The system includes an electron gun, deflection coils, and focusing optics, enabling the direct writing of intricate patterns with resolutions reaching sub-10 nm scales. This technique offers exceptional precision and flexibility for fabricating nanoscale devices but is limited by high costs and time-consuming serial processing.

#### 2.1.3. Three-Dimensional Printing Technologies

While lithographic techniques excel in fabricating high-resolution micro- and nanoscale patterns, they are often limited by their two-dimensional nature and reliance on specialized facilities. To overcome these constraints, 3D printing technologies have emerged as a complementary approach, enabling the construction of intricate three-dimensional architectures with tailored mechanical and biological properties. Unlike lithography, which is predominantly subtractive or pattern-transfer-based, 3D printing follows an additive manufacturing paradigm, allowing for greater design versatility and the direct fabrication of complex, patient-specific biomedical structures. By integrating lithography with 3D printing, researchers can harness the strengths of both techniques—combining the precision of nanoscale patterning with the structural complexity achievable through additive manufacturing—leading to innovative applications in tissue engineering, organ modeling, and personalized medicine.

Three-dimensional printing, also known as additive manufacturing, has become a transformative technology in biomedical engineering [38]. By building structures layer by layer, 3D printing offers unparalleled flexibility and precision in fabricating complex geometries. In healthcare applications, it enables the creation of patient-specific implants, tissue-engineered scaffolds, and drug delivery systems [39]. Two prominent methods for achieving high-resolution 3D printing include stereolithography (SLA) and two-photon polymerization.

#### 2.1.4. Stereolithography (SLA)

Stereolithography (SLA) is one of the earliest 3D printing technologies and remains a widely used technique for fabricating micro- and nano-architectures. SLA employs a light source, typically ultraviolet (UV) or laser, to cure liquid photopolymer resins layer by layer, creating highly precise structures [40]. In SLA, a vat of liquid photopolymer resin is exposed to a focused light beam that selectively solidifies the resin. The process begins with a build platform submerged slightly below the resin surface. As the light beam traces the desired pattern, the resin polymerizes, forming a solid layer. The platform then lowers incrementally, allowing the subsequent layers to be built on top [41]. SLA is extensively used in the fabrication of tissue-engineered scaffolds due to its ability to produce intricate geometries with controlled porosity and mechanical properties. For instance, SLA has been employed to create bone scaffolds with hierarchical pore structures that mimic native bone tissue. Additionally, SLA enables the production of microfluidic devices with precise channel geometries, facilitating applications in organ-on-a-chip systems and diagnostic assays. The key advantage of SLA is its high resolution, capable of achieving feature sizes as small as 50 microns It also allows for the use of a wide range of photopolymer resins, including those functionalized with bioactive molecules. However, SLA has limitations, including the need for post-processing to remove uncured resin and potential cytotoxicity of photopolymer residues [42]. Future advancements in SLA aim to enhance biocompatibility by developing novel bioresins that are both biocompatible and biodegradable. Combining SLA with other fabrication methods, such as inkjet printing, is also being explored to create hybrid structures for multifunctional biomedical devices.

Table 2 presents a comparative analysis of various fabrication techniques used to create micro- and nano-architectures, each offering distinct advantages and challenges depending on the application. Lithography-based methods, such as photolithography and electron beam lithography (e-beam), are renowned for their high precision and resolution, enabling the creation of intricate structures at the nanoscale, making them ideal for applications in microelectronics and sensors. However, these techniques are limited by high costs, complex setups, and the need for flat surfaces, restricting their broader applicability. In contrast, 3D printing technologies, including stereolithography (SLA) and two-photon polymerization, offer more flexibility and customization in fabricating complex 3D structures, making them highly suitable for applications like tissue engineering and medical device prototyping. However, the resolution of 3D printing methods is often lower than that of lithography, and issues such as limited material choices and slow fabrication speeds remain challenges. Self-assembly techniques, such as molecular self-assembly and block copolymer self-assembly, provide a more scalable, low-cost approach to fabricating nanoscale structures. These methods rely on the natural tendency of molecules or particles to organize into ordered patterns, offering efficient, energy-saving processes. Despite their advantages, these techniques face challenges in controlling the large-scale organization of structures and are often slower compared to lithographic approaches. Overall, the choice of fabrication technique depends on the specific requirements of the application, such as the desired resolution, material compatibility, and scalability, making it crucial to carefully assess each method’s strengths and limitations for optimal results in biomedical engineering applications.

#### 2.1.5. Two-Photon Polymerization

Two-photon polymerization (TPP) is a cutting-edge 3D printing technique that achieves nanoscale precision by utilizing the nonlinear absorption of femtosecond laser pulses. TPP is especially suited for fabricating highly detailed structures, such as nanostructured scaffolds and biosensors [43]. TPP relies on the simultaneous absorption of two photons in a tightly focused laser beam. The energy from these photons initiates polymerization in a localized region of the photopolymer resin. Since the polymerization occurs only at the focal point, TPP achieves a sub-diffraction-limited resolution, enabling the fabrication of features as small as 100 nm. TPP has been used to fabricate intricate scaffolds for neural tissue engineering, mimicking the complex architecture of the extracellular matrix [44]. It also facilitates the development of micro- and nanofluidic devices for single-cell analysis and molecular diagnostics. Additionally, TPP is employed to produce functional micromechanical devices, such as microrobots for targeted drug delivery. The major advantage of TPP is its exceptional resolution, enabling the creation of complex 3D structures at the nanoscale. However, the process is time-intensive and requires specialized equipment, making it less suitable for high-throughput applications [45]. Emerging trends in TPP include the development of faster laser scanning techniques and the integration of TPP with bioprinting technologies to fabricate hybrid bio-architectures. Advances in photopolymer chemistry are also expected to expand the range of materials compatible with TPP.

### 2.2. Self-Assembly Techniques

Self-assembly refers to the spontaneous organization of molecules or nanoscale building blocks into ordered structures without external guidance. This process harnesses weak intermolecular forces, such as van der Waals interactions, hydrogen bonding, and hydrophobic effects [46]. Self-assembly offers a cost-effective and scalable approach for fabricating micro- and nano-architectures, making it highly relevant in biomedical engineering.

#### 2.2.1. Molecular Self-Assembly

Molecular self-assembly involves the organization of individual molecules into ordered structures through non-covalent interactions. Examples include micelles, vesicles, and hydrogels, which are widely used in drug delivery and tissue engineering. Molecular self-assembly has been instrumental in developing drug delivery systems, such as liposomes and polymeric micelles, which encapsulate therapeutic agents and release them in response to specific stimuli [47]. For instance, self-assembled hydrogels have been used as injectable scaffolds for tissue regeneration, offering tunable mechanical properties and bioactivity. Additionally, self-assembled nanostructures, such as DNA origami, are being explored for precision medicine and diagnostics. The main advantage of molecular self-assembly is its simplicity and versatility. However, the stability of self-assembled structures can be influenced by environmental conditions, such as pH and ionic strength, which may limit their practical applications [48]. Future developments aim to enhance the stability and functionality of self-assembled materials by incorporating dynamic bonds or stimuli-responsive elements. Integrating self-assembly with top-down fabrication techniques also holds promise for creating hierarchical structures with enhanced properties.

#### 2.2.2. Block Copolymer Self-Assembly

Block copolymer self-assembly utilizes the phase separation of chemically distinct polymer blocks to form nanostructures with well-defined morphologies. Block copolymers have been used to fabricate nanostructured membranes for controlled drug release and biosensing [49]. Additionally, these materials have been employed to create nanoporous scaffolds for tissue engineering, providing enhanced cell attachment and nutrient diffusion. Block copolymer self-assembly offers precise control over morphology and size, enabling the creation of structures with tailored properties. However, achieving uniformity over large areas and scalability remain significant challenges [50]. Advancements in block copolymer chemistry aim to enable the formation of more complex morphologies and enhance scalability. Combining block copolymer self-assembly with external stimuli, such as magnetic or electric fields, is also being explored to guide the assembly process.

## 3. Applications in Therapeutics

Advancements in micro- and nano-architectures have significantly impacted therapeutic applications, particularly in drug delivery, tissue engineering, and implantable devices. These innovations address critical challenges in achieving site-specific action, prolonged therapeutic efficacy, and biocompatibility.

### 3.1. Drug Delivery Systems

Efficient drug delivery is fundamental to modern medicine, ensuring optimal therapeutic outcomes while minimizing side effects. Micro- and nano-architectures have emerged as transformative tools in designing advanced drug delivery systems (DDSs). These technologies allow for precise control over drug release profiles and targeted delivery to diseased tissues.

#### 3.1.1. Controlled Release Mechanisms

Controlled drug release systems are designed to maintain consistent therapeutic drug levels over an extended period. Such systems rely on micro- and nano-architectures that modulate the release rate through diffusion, degradation, or osmotic pressure.

##### Mechanisms of Controlled Release

**Diffusion-Controlled Systems**: These rely on the encapsulation of drugs within porous polymers or hydrogel matrices. Drugs diffuse gradually through the matrix, offering sustained release. For instance, poly(lactic-co-glycolic acid) (PLGA)-based microparticles have been widely used to deliver chemotherapeutic agents in cancer therapy [51]. PLGA (poly(lactic-co-glycolic acid)) microparticles are commonly used in controlled drug delivery systems due to their biodegradability, biocompatibility, and ability to encapsulate a wide variety of drugs [52]. In diffusion-controlled systems, PLGA microparticles release the encapsulated drug through a process governed by the diffusion of the drug from the polymer matrix to the surrounding environment. The drug diffuses from areas of high concentration (inside the microparticle) to low concentration (outside the microparticle) over time. PLGA microparticles behave as diffusion-controlled release systems because their porous nature allows for gradual and sustained drug release [53]. The rate of drug release is influenced by several factors, including the size of the microparticles, the molecular weight of the polymer, and the drug’s physicochemical properties. For example, in cancer therapy, PLGA microparticles are used to deliver chemotherapeutic agents like paclitaxel, doxorubicin, or methotrexate [54]. These drugs are encapsulated in the polymer matrix, which breaks down over time via hydrolytic degradation. As PLGA degrades, it releases the drug in a controlled manner, often over weeks to months, depending on the formulation and environmental conditions. This prolonged release minimizes the need for frequent drug administration, reduces side effects, and enhances therapeutic efficacy by maintaining a consistent drug concentration at the target site.

**Biodegradable Polymers**: Biodegradable scaffolds, such as those made from chitosan or gelatin, release drugs as they degrade under physiological conditions [55]. This approach has shown great promise in post-surgical applications, where localized drug delivery reduces infection risks. Biodegradable polymers, such as chitosan and gelatin, have gained significant attention for their ability to provide controlled drug release through their gradual degradation under physiological conditions [55]. These polymers are widely used in tissue engineering and drug delivery applications due to their biocompatibility and biodegradability. For example, chitosan-based scaffolds have been utilized for localized drug delivery in wound healing and post-surgical applications, where they release antibiotics or anti-inflammatory agents to reduce infection risks and promote tissue regeneration [56]. Similarly, gelatin-based scaffolds have been explored for the sustained release of growth factors or antimicrobial agents in surgical wound sites, aiding in tissue repair and minimizing post-operative infections [57]. The degradation of these polymers over time allows for continuous drug release, enhancing therapeutic efficacy and reducing the need for frequent drug administration. These approaches are particularly beneficial in reducing infection risks in post-surgical settings, as they provide localized, sustained drug concentrations at the site of injury or surgery.

**Osmotic Systems**: Micron-scale osmotic pumps, integrated with nanovalves, are advanced drug delivery systems designed to release therapeutics in a controlled and sustained manner [58]. These pumps operate based on the osmotic gradient, where water is drawn into the pump chamber, creating pressure that drives the drug through a micro- or nanoscale valve. The integration of nanovalves, which can be triggered by external stimuli (such as pH, temperature, or enzymatic activity), adds an extra layer of control, ensuring precise dosing over time. This mechanism allows for the release of drugs at a constant rate, minimizing fluctuations in drug concentration that could lead to side effects or suboptimal therapeutic outcomes. Micron-scale osmotic pumps are particularly valuable in applications requiring long-term, continuous drug delivery, such as chronic disease management or post-operative care. The use of nanovalves enhances the specificity and responsiveness of the system, allowing for personalized dosing regimens that can adapt to changing conditions in the body. These pumps have shown promise in applications ranging from cancer therapy, where targeted drug delivery can be maintained, to the management of chronic conditions like diabetes, where insulin release can be fine-tuned to match the patient’s needs.

Controlled release DDSs have been employed for managing chronic diseases like diabetes, cardiovascular conditions, and cancer. By minimizing systemic exposure, these systems reduce toxicity while improving patient compliance. Ensuring uniform drug distribution and scaling up manufacturing remain critical challenges. Advances in smart materials and multi-compartment systems are likely to enhance the sophistication of controlled DDSs.

#### 3.1.2. Targeted Delivery Strategies

Targeted drug delivery systems are designed to deliver therapeutics directly to diseased tissues while sparing healthy ones. This precision is achieved through functionalization of micro- and nanoscale carriers with ligands or antibodies [59].

**Passive Targeting**: Utilizes the enhanced permeability and retention (EPR) effect, particularly effective in cancer therapy where leaky vasculature allows nanoparticles to accumulate in tumors [60].

**Active Targeting**: Relies on surface modifications with targeting moieties, such as antibodies, peptides, or aptamers [61]. For example, folic acid-conjugated nanoparticles have shown specificity for cancer cells overexpressing folate receptors.

Targeted delivery systems are transforming fields like oncology, where nano-drug carriers deliver chemotherapeutics directly to tumors, reducing off-target effects. Additionally, these systems are being applied in gene therapy, where nanoscale carriers deliver nucleic acids specifically to diseased cells [62]. Ensuring stability during circulation and efficient intracellular delivery remain significant challenges. Future strategies involve hybrid delivery systems that combine targeting with stimuli-responsive features to enhance efficacy.

### 3.2. Tissue Engineering and Regenerative Medicine

**Figure 2:**
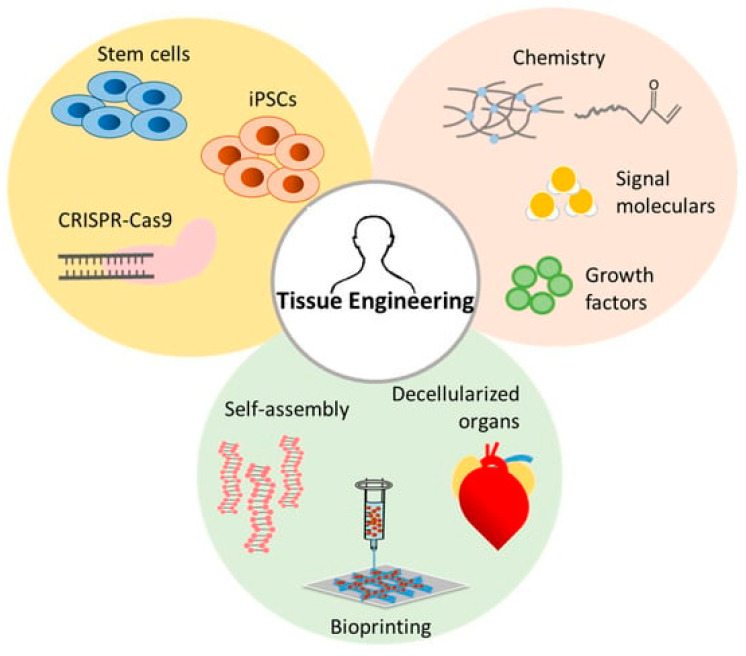
Overview of advances in tissue engineering. Copyright permission from references [63], ccby 4.0. https://doi.org/10.3390/nano10050887.

Micro- and nano-architectures have revolutionized tissue engineering, providing scaffolds and materials that mimic the extracellular matrix (ECM). These systems support cellular growth, differentiation, and tissue regeneration. Figure 2 provides a comprehensive overview of recent advancements in tissue engineering, highlighting key strategies and innovations that have propelled the field forward [63]. Tissue engineering integrates biomaterials, cellular engineering, and biofabrication techniques to develop functional tissue constructs for regenerative medicine and transplantation. Advances in scaffold design, including the use of biodegradable polymers, hydrogels, and nanocomposites, have significantly improved cell adhesion, proliferation, and differentiation. Additionally, 3D bioprinting has emerged as a transformative tool, enabling the precise spatial arrangement of cells and biomaterials to mimic native tissue architecture. The integration of stem cells and growth factors further enhances the regenerative potential of engineered tissues, while stimuli-responsive materials provide dynamic environments that adapt to physiological conditions. Despite these advancements, challenges such as vascularization, immune compatibility, and large-scale manufacturing remain critical areas of ongoing research to bring engineered tissues closer to clinical applications.

#### 3.2.1. Scaffolds for Cellular Interactions

Scaffolds serve as structural frameworks that facilitate cell attachment, proliferation, and differentiation. Advanced fabrication techniques have enabled the design of scaffolds with hierarchical porosity and bioactive cues.

**Porosity and Interconnectivity**: Highly porous scaffolds enhance nutrient diffusion and waste removal, critical for tissue survival [64].

**Bioactive Materials**: Incorporation of growth factors or ECM-mimicking peptides promotes cellular signaling and tissue regeneration. For example, hydroxyapatite-based scaffolds are widely used in bone tissue engineering [65].

Scaffolds have been successfully employed in regenerating bone, cartilage, and skin tissues. Emerging applications include neural scaffolds that guide axonal growth in spinal cord injuries. Achieving precise control over scaffold degradation rates and integrating vascularization remain key challenges [66]. Four-dimensional scaffolds, which dynamically adapt to the physiological environment, represent an exciting frontier.

#### 3.2.2. Stimuli-Responsive Materials

Stimuli-responsive materials dynamically alter their properties in response to environmental triggers, such as pH, temperature, or mechanical forces, making them valuable for biomedical applications. These materials enable controlled drug release, adaptive scaffolds, and responsive biosensors, enhancing therapeutic precision and patient outcomes.

Types of Stimuli-Responsive Materials:

**pH-Responsive Polymers:** These polymers release drugs or bioactive molecules in response to pH variations, particularly useful in tumor-targeted therapies where the acidic microenvironment triggers drug release [67].

**Shape-Memory Polymers:** Used in tissue engineering, these polymers regain their original shape under specific stimuli, facilitating minimally invasive implantation and promoting regeneration [68].

Stimuli-responsive hydrogels have shown promise in wound healing by releasing antimicrobial agents in response to infection-related pH changes. Additionally, integrating these materials into bioinks for 3D bioprinting creates dynamic tissue scaffolds that respond to physiological cues, enhancing cellular integration [69]. A major research focus is developing multi-responsive materials that simultaneously react to multiple stimuli, improving their adaptability for complex biological systems. To provide a comprehensive overview, Table 3 summarizes various stimuli-responsive materials, their mechanisms, biomedical applications, advantages, and limitations. These materials exhibit unique responsiveness to stimuli, such as pH, temperature, light, and magnetic or electric fields, making them highly versatile for drug delivery, tissue engineering, and biosensing. Stimuli-responsive materials continue to evolve, with enzyme-responsive hydrogels emerging as a crucial category due to their specificity in biological microenvironments. These materials undergo structural or functional transformations in response to enzymatic activity, enabling controlled drug release, targeted degradation, or dynamic scaffold remodeling. In tissue engineering, enzyme-sensitive hydrogels can facilitate localized and on-demand degradation, promoting cell infiltration and integration within engineered constructs. Similarly, in drug delivery applications, these materials offer precise therapeutic control by responding to overexpressed enzymes in diseased tissues, such as matrix metalloproteinases in cancer or inflammatory conditions. By incorporating enzyme-responsive materials into the broader landscape of stimuli-responsive systems, we provide a more comprehensive understanding of their adaptability and potential in biomedical applications. Enzyme-responsive materials represent a crucial advancement in stimuli-responsive systems, leveraging enzymatic activity in physiological conditions to achieve targeted therapeutic effects [70]. These materials undergo structural or chemical transformations in response to specific enzymes, making them particularly useful for cancer therapy, where tumor-associated enzymes, such as matrix metalloproteinases (MMPs), trigger controlled drug release within the tumor microenvironment. Additionally, enzyme-responsive hydrogels have been explored for infection-responsive wound dressings, where bacterial enzymes initiate antimicrobial agent release at infection sites, enhancing localized treatment efficacy. For instance, recent studies have demonstrated the potential of enzyme-sensitive nanocarriers for precision drug delivery, further highlighting their biomedical significance [70].

### 3.3. Therapeutic Implants and Devices

Therapeutic implants and devices have benefited greatly from advancements in micro- and nano-architectures. These technologies enable the design of biocompatible, long-lasting implants with enhanced therapeutic functionality [79].

**Drug-Eluting Implants**: Incorporating nano-porous coatings enables controlled drug release, improving therapeutic efficacy and reducing side effects. For example, Sirolimus-eluting coronary stents (~50–200 nm porous coatings) release anti-proliferative drugs to prevent restenosis in cardiovascular patients [80]. Additionally, antibiotic-eluting bone implants (e.g., gentamicin-loaded titanium implants with nano-porous coatings) reduce post-surgical infections in orthopedic procedures.

**Sensors and Actuators**: Nano-functionalized biosensors allow the real-time monitoring of physiological parameters. For instance, nano-engineered glucose sensors (~10–50 nm nanowire-based transducers) improve sensitivity in diabetes management [81]. Similarly, nano-actuators in drug delivery systems, such as magnetically controlled nanoparticles (~50–200 nm), facilitate targeted drug release for cancer therapy.

**Orthopedic Implants**: The surface nano-topography of orthopedic implants plays a crucial role in bone integration. Titanium implants with nano-patterned surfaces (~10–100 nm roughness features) enhance osteoblast adhesion, accelerating osseointegration [82]. Additionally, hydroxyapatite-coated nanostructured implants (~50–500 nm layers) mimic the natural bone environment, improving implant longevity and reducing failure rates.

Balancing mechanical strength with biocompatibility is a key challenge. Future implants may incorporate self-healing materials and integrated biosensors for personalized healthcare.

## 4. Applications in Diagnostics

### 4.1. Biosensing and Detection Technologies

Biosensors have become integral tools in modern diagnostics due to their ability to provide a rapid, sensitive, and cost-effective detection of biomarkers, pathogens, and disease-related molecules. The integration of nano-biosensors into diagnostic applications is a significant breakthrough, as they offer enhanced sensitivity and specificity over traditional biosensors. Nano-biosensors operate through the transduction of a biological signal into a measurable physical or chemical signal, facilitated by the unique properties of nanomaterials (such as high surface-area-to-volume ratios, quantum effects, and surface plasmon resonance). These biosensors have demonstrated substantial advancements in detecting disease biomarkers, like DNA, RNA, proteins, and metabolites at ultra-low concentrations, making them ideal for early-stage disease detection and personalized medicine applications [83]. Moreover, the incorporation of point-of-care (POC) devices into these biosensing platforms is revolutionizing healthcare by enabling rapid diagnostics in resource-limited settings. POC devices, often integrated with nano-biosensors, provide portable and user-friendly systems for onsite diagnostic testing, such as glucose monitoring, cancer biomarker detection, and infectious disease diagnostics. These devices leverage microfluidic technologies, portable electronics, and real-time data analytics, which allow patients to receive immediate diagnostic results without needing to go to a laboratory, significantly reducing diagnostic time and healthcare costs.

Key Features of Point-of-Care (POC) Devices: POC devices are designed to be compact, user-friendly, and highly sensitive, ensuring that non-specialists can perform diagnostic tests with minimal training. These devices prioritize rapid response times, often delivering results within minutes, which is crucial for early disease detection and timely clinical interventions. Miniaturization and portability enable POC devices to be used in remote or resource-limited settings, making healthcare more accessible, particularly in developing regions. Additionally, integration with microfluidics allows for the precise handling of small sample volumes, reducing reagent consumption and overall costs. Another key feature is real-time connectivity, with many POC devices incorporating wireless data transmission and smartphone compatibility, enabling healthcare providers to monitor patient data remotely. The use of multiplex detection platforms further enhances diagnostic capabilities by allowing the simultaneous detection of multiple biomarkers within a single test, improving efficiency and clinical decision-making. Finally, advancements in AI-powered POC systems are improving diagnostic accuracy by enabling an automated interpretation of test results, reducing human error, and facilitating personalized treatment strategies.

The U.S. Food and Drug Administration (FDA) has approved various medical devices and personalized medicine approaches across multiple categories. Here are some notable examples:

1. Implants: Breast Implants: The FDA has approved both saline-filled and silicone gel-filled breast implants for breast augmentation and reconstruction. Saline-filled implants are approved for augmentation in women aged 18 years or older, while silicone gel-filled implants are approved for those aged 22 years or older. These implants are also utilized in revision surgeries to correct or improve the results of original procedures [84].

Motiva Implants: In September 2024, the FDA approved Motiva Implants, marking the first new breast implant premarket approval since 2013. These implants are known for their natural feel and improved health features, utilizing SmoothSilk silicone shells to reduce complications, like capsular contracture [85].

2. Point-of-Care (POC) Devices: Over-the-Counter Glucose Monitors: In March 2024, the FDA approved the Dexcom Stelo, the first over-the-counter continuous glucose monitoring system. Designed for individuals with type 2 diabetes or prediabetes not using insulin, this wearable sensor provides real-time glucose readings, empowering users to manage their condition effectively [86].

3. Wearable Sensors: Apple Watch Sleep Apnea Detection: Apple received FDA approval for a sleep apnea detection feature in its watchOS 11 software, compatible with Apple Watch Series 9, Ultra 2, and Series 10. This feature monitors wrist movements during sleep to identify disruptions in respiratory patterns, aiding in the early detection of sleep apnea [87].

4. Personalized Medicine: Continuous Glucose Monitoring Sensors: FDA-approved continuous glucose monitoring sensors have been integrated into wearable devices, facilitating continuous health monitoring and enabling personalized medicine approaches. These sensors play a crucial role in early disease detection and improved health outcomes by providing real-time data on glucose levels. These FDA approvals highlight the advancements in medical devices and personalized medicine, offering patients innovative solutions for health monitoring and management.

**Figure 3:**
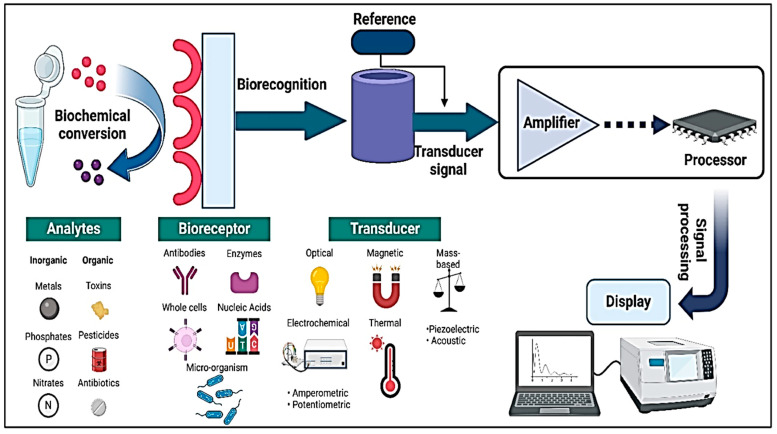
Essential elements of biosensors. Copyright permission from ref no [88]. https://doi.org/10.3390/s24165143.

Figure 3 illustrates the essential components of biosensors, which are highly sophisticated analytical devices designed for the rapid and selective detection of biological or chemical substances [88]. A typical biosensor consists of three primary elements: a bioreceptor, a transducer, and a signal processor. The bioreceptor, which can be an enzyme, antibody, nucleic acid, or aptamer, specifically interacts with the target analyte, ensuring high selectivity. The transducer converts this biological interaction into a measurable signal, commonly using optical, electrochemical, or piezoelectric mechanisms. Finally, the signal processor amplifies and interprets the signal, displaying the results in a user-friendly format. These components work synergistically to enable the real-time, high-sensitivity detection of biomarkers, pathogens, or environmental pollutants, making biosensors invaluable tools in medical diagnostics, environmental monitoring, and food safety applications. Ongoing advancements in nanotechnology, microfluidics, and AI-driven data processing continue to enhance the efficiency and miniaturization of biosensors, paving the way for next-generation point-of-care and wearable diagnostic devices.

#### 4.1.1. Nano-Biosensors for Disease Detection

Nano-biosensors have emerged as one of the most promising diagnostic tools due to their ability to detect diseases at the molecular level with exceptional sensitivity. By incorporating nanoparticles, like gold, silver, or carbon-based materials (e.g., graphene and carbon nanotubes), nano-biosensors exhibit enhanced surface interaction, leading to stronger signal amplification. These materials can be tailored for specific biomolecular targets, enabling the early detection of diseases, such as cancer, infectious diseases, and neurodegenerative disorders. For example, gold nanoparticle-based biosensors have been utilized for the detection of circulating tumor DNA (ctDNA) and microRNAs, which are critical biomarkers for early-stage cancer detection [89]. Graphene-based sensors provide high electrical conductivity, making them excellent for electrochemical biosensing, where small changes in the target biomolecule concentration lead to measurable shifts in the electrical properties. The capability of nano-biosensors to provide highly sensitive, rapid, and cost-effective disease detection is transforming diagnostics, with applications in detecting a wide range of diseases from diabetes to infectious diseases like COVID-19.

#### 4.1.2. Integration with Point-of-Care Devices

The integration of nano-biosensors with point-of-care (POC) devices is one of the most significant advancements in medical diagnostics. These systems combine the benefits of nanoscale sensitivity with the accessibility and convenience of portable diagnostic devices, enabling real-time monitoring and analysis at the patient’s bedside or in remote settings. For example, portable glucose monitors with integrated nano-biosensors provide continuous monitoring for diabetes patients. Similarly, lateral flow immunoassays have been enhanced with gold nanoparticles for detecting infectious agents, like viruses and bacteria, at low concentrations, providing quick results for conditions such as HIV, Zika virus, and COVID-19 [90]. By minimizing the need for centralized lab-based testing, POC devices integrated with nano-biosensors significantly reduce diagnostic time and improve patient outcomes, particularly in urgent care and developing regions where access to specialized labs is limited.

### 4.2. Imaging and Contrast-Enhancing Agents

**Imaging techniques**, such as magnetic resonance imaging (MRI), computed tomography (CT), and ultrasound, are crucial for the non-invasive diagnostic imaging of anatomical structures and physiological conditions. However, the inherent limitations of these techniques, such as low spatial resolution or poor sensitivity, can hinder their ability to detect diseases at early stages. This challenge has been addressed through the development of contrast-enhancing agents, particularly nanomaterials, which can improve the signal intensity and resolution of these imaging modalities. Nanoparticles such as iron oxide nanoparticles (for MRI) and gold nanoparticles (for CT and ultrasound) are often used as contrast agents because of their unique physical properties, including high atomic number, biocompatibility, and ability to interact with electromagnetic fields [91]. In MRI, superparamagnetic nanoparticles, such as iron oxide, are utilized to improve contrast, enabling the clearer imaging of tissues, such as tumors and blood vessels. Gold nanoparticles enhance X-ray imaging by increasing the absorption of X-rays, making them particularly valuable in CT scans. Furthermore, nanoparticles can be engineered with targeting ligands, such as antibodies or peptides, to selectively bind to disease-specific biomarkers, enabling molecular imaging that allows visualization of specific disease processes at the cellular and molecular levels. These targeted imaging agents enhance the sensitivity of imaging techniques, facilitating the early detection, accurate diagnosis, and real-time monitoring of disease progression. The effectiveness of gold and iron oxide nanoparticles as imaging and contrast-enhancing agents stems from their distinct physicochemical properties. Gold nanoparticles (AuNPs) have a high atomic number (Z = 79), which results in strong X-ray attenuation, making them ideal for CT imaging. Their tunable size and surface plasmon resonance properties allow precise control over optical absorption and scattering, enhancing signal clarity in both CT and photoacoustic imaging. Additionally, gold’s excellent biocompatibility and ease of functionalization with biomolecules enable targeted imaging with minimal toxicity. On the other hand, iron oxide nanoparticles (IONPs) exhibit superparamagnetic behavior at the nanoscale, meaning they strongly enhance MRI contrast by shortening transverse (T2) and longitudinal (T1) relaxation times of protons in surrounding water molecules. This effect leads to significant signal enhancement, allowing for a clearer differentiation of soft tissues, blood vessels, and pathological regions, such as tumors. Moreover, both AuNPs and IONPs can be modified with surface coatings (e.g., PEG, silica, or antibodies) to prolong circulation time, improve bioavailability, and target specific tissues or disease biomarkers, thereby enhancing diagnostic accuracy and facilitating early disease detection.

### 4.3. Lab-on-a-Chip and Microfluidic Platforms

Lab-on-a-chip (LOC) and microfluidic platforms represent miniaturized, integrated systems that combine multiple laboratory functions onto a single chip, typically on the scale of a few millimeters to centimeters. These systems have revolutionized the field of diagnostics by enabling highly efficient, rapid, and low-cost assays for disease detection. LOC platforms are capable of performing complex biological analyses, such as blood analysis, DNA sequencing, and protein assays, within a small, portable device. Microfluidic systems utilize small channels and precise fluid flow control to manipulate and analyze small volumes of samples with minimal reagent consumption. By incorporating nano-biosensors into these microfluidic devices, LOC systems can detect and analyze biomarkers at the molecular level in real time. This integration has led to the development of point-of-care diagnostic devices capable of detecting a wide range of diseases, ranging from infectious diseases to cancer. The advantages of LOC systems include reduced sample and reagent volumes, enhanced speed, portability, and the potential for high-throughput screening. Moreover, microfluidic platforms can be designed for multiplexed assays, allowing for the simultaneous detection of multiple biomarkers in a single sample, thus enabling comprehensive diagnostic capabilities [92]. These systems have the potential to democratize access to diagnostics, particularly in low-resource settings, where traditional laboratory-based diagnostic tools are not feasible.

Table 4 provides a detailed comparative analysis of key diagnostic applications, focusing on Biosensing and Detection Technologies, Imaging and Contrast-Enhancing Agents, and Lab-on-a-Chip and Microfluidic Platforms. Each application offers distinct advantages and challenges, making them suitable for different diagnostic scenarios. Nano-biosensors, integrated with point-of-care devices, enable the rapid, sensitive detection of disease biomarkers, offering the promise of on-site diagnostics in resource-limited settings. However, their performance can be influenced by non-specific binding and the need for precise functionalization. Imaging and contrast-enhancing agents, such as nanoparticles and quantum dots, provide high-resolution imaging with enhanced contrast, aiding early disease detection. Despite their potential, issues like toxicity and photobleaching remain limiting factors. Lastly, Lab-on-a-chip and microfluidic platforms present miniaturized, high-throughput solutions for diagnostics, offering portability and reduced reagent consumption. While they offer significant advantages, the challenges of device scalability and integration of multiple functions remain. Each technology continues to evolve, with ongoing efforts to overcome their limitations and enhance their clinical utility.

## 5. Design Parameters for Micro- and Nano-Architectures

The successful integration of micro- and nano-architectures into biomedical applications relies heavily on several critical design parameters. These parameters dictate not only the performance of these materials in vivo, but also their long-term sustainability, scalability, and safety. In this section, we discuss the essential considerations that must be addressed in designing micro- and nano-architectures for biomedical applications, including biocompatibility and safety, mechanical properties and resilience, and scalability and cost-effectiveness.

### 5.1. Biocompatibility and Safety Considerations

Biocompatibility is one of the most fundamental design parameters for any material intended for use in the human body. It refers to the ability of a material to coexist with living tissue without eliciting any adverse immune or inflammatory response. The biological interaction between engineered micro- and nano-architectures and living cells or tissues must be well understood and carefully optimized to avoid toxic or inflammatory reactions. For instance, nanomaterials, such as gold nanoparticles, carbon nanotubes, and graphene, have shown remarkable promise in biomedical applications due to their unique properties, such as high surface area, tunable surface chemistry, and ease of functionalization. However, their interaction with biological systems can result in unwanted effects like cytotoxicity, immune system activation, or unwanted accumulation in organs such as the liver or spleen [99]. To address these challenges, researchers focus on surface modifications of nanomaterials to enhance their biocompatibility. Coating nanomaterials with biologically relevant molecules, such as polyethylene glycol (PEG) or cell-adhesive peptides, can improve their stability and reduce immune recognition, enabling prolonged circulation in the bloodstream. Additionally, materials designed for drug delivery systems must ensure that degradation products are non-toxic and do not disrupt the surrounding tissues. Biodegradable materials, such as certain biopolymers and hydrogels, are favored in many biomedical applications because they naturally break down into non-toxic byproducts over time. Despite these advancements, the biocompatibility of new materials requires extensive in vitro and in vivo testing to ensure safety, particularly when considering chronic exposure or implantation in the human body.

### 5.2. Mechanical Properties and Resilience

The mechanical properties of micro- and nano-architectures are crucial for their performance in biomedical applications, particularly in tissue engineering and implantable devices. These properties include strength, elasticity, toughness, and resilience, which govern how a material behaves under various mechanical forces, such as compression, tension, and shear stress. For tissue engineering applications, the mechanical properties of scaffolds must closely mimic those of the natural tissues they are intended to replace or repair. For example, bone tissue requires scaffolds with high mechanical strength and stiffness, while soft tissues, like cartilage or skin, require materials with more flexibility and elasticity. Hydrogels and elastomers, as part of the soft materials family, are frequently employed for creating scaffolds with appropriate mechanical properties for soft tissue regeneration [100]. The resilience of these materials is particularly important in environments where dynamic mechanical forces are constantly at play, such as in implantable devices or electronic skin. In applications like wearable electronics or prosthetics, the materials must be able to withstand repeated deformation without degrading over time. Self-healing materials, which can recover their mechanical properties after damage, are also being explored in the context of bioelectronics to improve the longevity and reliability of devices exposed to mechanical stress [101]. Moreover, the mechanical interaction between the implanted material and the surrounding tissue plays a significant role in the integration of the device and its functional performance. Poor mechanical matching between the material and tissue can lead to chronic inflammation, implant rejection, or failure. Therefore, a balance between mechanical performance and biological response is key to the success of micro- and nano-architectures in medical applications.

### 5.3. Scalability and Cost-Effectiveness

The scalability and cost-effectiveness of producing micro- and nano-architectures are major factors that influence their transition from the laboratory to real-world clinical applications. While significant progress has been made in the fabrication of small-scale micro- and nanostructures, the scalability of these technologies remains a key challenge for mass production. Fabrication techniques, like lithography, 3D printing, and self-assembly, offer precise control at the micro- and nanoscales, but many of these methods are either costly or difficult to scale up for large-scale production. For example, electron beam lithography, though highly precise, is slow and expensive, making it impractical for mass production of large quantities of nano-devices [102]. In contrast, 3D printing technologies, particularly stereolithography (SLA) and two-photon polymerization, have shown promise in producing complex micro- and nano-architectures at relatively low costs, making them more suitable for scaling. However, the challenges of maintaining high resolution and consistent material properties during large-scale production need to be addressed. Moreover, the cost-effectiveness of materials is a critical consideration, particularly when designing diagnostic tools, drug delivery systems, or implantable devices for widespread use. The use of rare or expensive materials, such as gold nanoparticles, can significantly increase the cost of production. To address this, researchers are exploring alternative, cost-effective materials that still provide a high performance, such as carbon-based nanomaterials (e.g., graphene and carbon nanotubes), which offer a favorable combination of performance, cost, and availability. Additionally, the integration of automated manufacturing processes, such as roll-to-roll printing for the large-area production of nanostructures, could help reduce costs and improve scalability, thus making these technologies more accessible for widespread medical use. Ultimately, achieving both scalability and cost-effectiveness will be crucial for the commercial success and large-scale implementation of micro- and nano-architectures in biomedical engineering.

**Figure 4:**
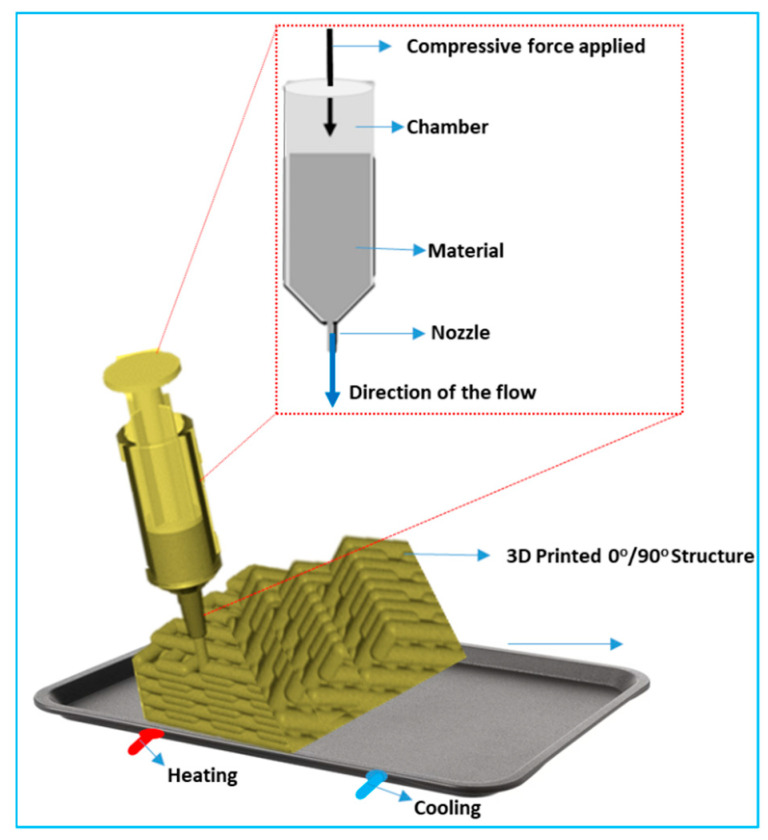
Schematic representation of 3D printing process.

Figure 4 depicts the 3D printing process, also known as additive manufacturing, which involves the layer-by-layer deposition of materials to create a three-dimensional object from a digital design. This technique allows for high precision in fabricating complex geometries and structures that are difficult to achieve with traditional manufacturing methods. The process begins with creating a digital model, typically using CAD (computer-aided design) software, which is then converted into machine-readable instructions (G-code). The material, which can range from polymers, metals, ceramics, or biomaterials, is fed into the printer and heated to a semi-liquid state (in the case of thermoplastic materials) or a photo-sensitive resin is cured (for stereolithography) as the printer deposits successive layers. Each layer is fused to the one below it, allowing for intricate, customized structures to be formed. This method is particularly valuable in biomedical engineering, where it can be used to create patient-specific implants, scaffolds for tissue engineering, and drug delivery devices with precise micro- and nanoscale architectures. The ability to design and fabricate scaffolds that closely mimic the natural extracellular matrix (ECM) offers significant improvements in tissue regeneration and cellular growth. Moreover, 3D printing allows for the integration of multiple materials in a single construct, enabling the development of multifunctional devices that combine structural support, biochemical cues, and therapeutic agents. As the technology continues to advance, it promises further innovations in personalized medicine, providing solutions tailored to individual patient needs and improving the efficacy of biomedical treatments.

Table 5 presents a comparative analysis of the key design parameters for micro- and nano-architectures in biomedical applications, focusing on biocompatibility and safety, mechanical properties and resilience, and scalability and cost-effectiveness. Biocompatibility remains a critical factor, as materials must interact harmoniously with biological systems to avoid toxicity and immune response. Surface modifications, such as functionalization with polyethylene glycol (PEG), can enhance material compatibility and extend the circulation time in the body, but concerns about long-term stability and potential toxicity from degradation products persist. In terms of the mechanical properties, materials must closely mimic the mechanical properties of native tissues to ensure proper integration and function, particularly in tissue engineering and implantable devices. The development of self-healing materials adds a layer of resilience, improving longevity and minimizing the need for replacements. However, matching the mechanical properties of native tissues while maintaining the material performance over time remains challenging. Lastly, scalability and cost-effectiveness are key for the practical application of these technologies. Techniques like 3D printing and roll-to-roll processing enable the large-scale production of complex structures, but the cost of materials, particularly rare ones, like gold, and limitations in fabricating high-precision features at scale, present significant barriers. Therefore, achieving an optimal balance between biocompatibility, mechanical strength, and scalability is crucial for advancing the clinical implementation of micro- and nano-architectures in biomedical engineering.

## 6. Challenges and Limitations

While micro- and nano-architectures hold immense promise in revolutionizing biomedical engineering, their integration into clinical settings faces several challenges. These challenges are multi-faceted and encompass issues related to clinical translation, long-term stability and performance, and regulatory and ethical considerations. In this section, we discuss these challenges in detail and explore the ongoing efforts to address them.

### 6.1. Barriers to Clinical Translation

The transition from laboratory research to clinical application is one of the most significant challenges in biomedical engineering, particularly for micro- and nano-architectures. Despite the promising results from preclinical studies, several barriers impede the clinical translation of these materials. One primary barrier is the complexity of these materials, which often requires highly specialized fabrication techniques. Nanofabrication processes, such as photolithography or electron beam lithography, offer exceptional precision but are expensive and difficult to scale for large-scale manufacturing. Moreover, the intricate fabrication steps often result in high production costs, making these materials economically unfeasible for mass clinical use. Another critical challenge is the biocompatibility and in vivo performance of these materials over long periods. While many micro- and nano-architectures have been shown to be biocompatible in vitro, their performance in living organisms is still a major concern. Issues, such as immune system activation, chronic inflammation, and implant rejection, can arise once these materials are implanted into the human body. Additionally, material degradation and the leakage of drug delivery systems over time are critical factors that must be addressed to ensure the safety and efficacy of these technologies in clinical settings. Long-term safety data, particularly for chronic implants or drug delivery systems, are still lacking, making the regulatory approval process more challenging. Furthermore, there is the issue of standardization in the production of micro- and nano-architectures. Variations in material properties between batches can lead to inconsistent outcomes, affecting both the clinical efficacy and patient safety. Ensuring reproducibility in manufacturing processes and developing uniform protocols for clinical applications are essential for overcoming these barriers.

### 6.2. Long-Term Stability and Performance

The long-term stability and performance of micro- and nano-architectures are pivotal for their practical application in biomedical settings. These materials must be able to maintain their functional properties over extended periods of time in the physiological environment. For instance, in drug delivery applications, nano-carriers must retain their structural integrity and controlled release properties throughout the treatment period without significant degradation or loss of therapeutic efficacy. In implantable devices or scaffolds for tissue engineering, long-term durability is essential to prevent mechanical failure or degradation that could disrupt tissue regeneration or lead to adverse effects. Material degradation is a particular concern, especially for biodegradable materials that are designed to break down over time within the body. While these materials are useful for drug delivery and tissue regeneration, their degradation rates must be carefully controlled to avoid premature failure or accumulation of harmful degradation products. Additionally, the mechanical properties of materials, such as strength, elasticity, and toughness, must remain consistent over time to ensure proper functioning, particularly in the case of load-bearing implants or electronic devices. The biological environment further complicates the issue of stability, as enzymes, pH variations, and oxidative conditions in the body can accelerate material degradation. In bioelectronics and wearable devices, for instance, the constant mechanical wear and environmental exposure can degrade material properties over time, reducing device efficiency. To enhance long-term stability, researchers are focusing on developing self-healing materials, protective coatings, and robust encapsulation techniques that can mitigate degradation and ensure sustained performance.

### 6.3. Regulatory and Ethical Considerations

The regulatory landscape for micro- and nano-architectures in biomedical applications is complex and evolving. Regulatory agencies, such as the U.S. Food and Drug Administration (FDA) and the European Medicines Agency (EMA), have set stringent guidelines for the approval of medical devices and therapies. These agencies require comprehensive safety and efficacy data to ensure that new materials and devices do not pose a risk to patients. However, the novelty and complexity of micro- and nano-architectures present challenges in meeting these regulatory requirements. Standard protocols for assessing the safety of nanomaterials are still being developed, and there is an ongoing debate about the need for specialized testing for nanotoxicity and bioaccumulation. In recent years, regulatory progress has been made in addressing ethical concerns and streamlining approval pathways for nano-enabled personalized medicine. The FDA has established the Nanotechnology Regulatory Science Research Plan, focusing on the risk assessment, biocompatibility, and long-term safety of nanomaterials in healthcare applications. Additionally, the ISO 10993 standard for the biological evaluation of medical devices has been expanded to include considerations specific to nanoscale materials. Ethical concerns related to patient consent and transparency have also been integrated into regulatory frameworks, ensuring that patients receive comprehensive information regarding the risks and benefits of nano-enabled treatments. Furthermore, international collaborations, such as the OECD Working Party on Manufactured Nanomaterials (WPMN), are developing harmonized testing strategies to address bioaccumulation concerns and improve public trust in nanomedicine. Clinical trial designs for personalized medicine using nano-micro architectures are advancing to accommodate individualized therapeutic approaches. The FDA has supported adaptive clinical trial designs, which allow real-time modifications based on patient responses, improving the precision of nano-enabled therapies. These trials incorporate biomarker-based stratification, where patients are grouped based on molecular profiles, ensuring targeted drug delivery and maximizing therapeutic outcomes. Additionally, patient-derived organoids and microfluidic-based clinical trial models are being developed to assess the efficacy of nano-formulated drugs before human trials, reducing risks and accelerating regulatory approval. As regulatory frameworks continue to evolve, these advancements will facilitate the integration of micro- and nano-architectures into personalized medicine, ultimately improving patient outcomes while maintaining stringent safety and ethical standards.

## 7. Future Perspectives

The field of micro- and nano-architectures in biomedical engineering is rapidly evolving, with exciting innovations and trends shaping the future landscape. In this section, we discuss the emerging trends, the integration of micro- and nano-architectures with personalized medicine, and a proposed roadmap for the translational research. These developments promise to significantly impact the way we approach diagnostics, therapeutics, and healthcare delivery, addressing the existing challenges and providing new opportunities for precision medicine.

### 7.1. Emerging Trends and Innovations

Advancements in micro- and nano-architectures have led to the development of highly functional biomaterials with adaptive and precision-driven properties. One key trend is the integration of nanotechnology with smart materials, enabling personalized and real-time healthcare solutions. For instance, nano-enabled sensors are being integrated into wearable devices for continuous health monitoring and disease prevention. These sensors detect specific biomarkers at ultra-low concentrations, facilitating early diagnosis and individualized treatment. Additionally, nanorobotics is emerging as a transformative approach, with tiny autonomous robots capable of targeted drug delivery, tissue repair, and the removal of harmful substances at the cellular level [110]. Three-dimensional bioprinting is revolutionizing tissue engineering by allowing the fabrication of patient-specific, vascularized tissue scaffolds, providing a breakthrough in regenerative medicine and organ transplantation. Furthermore, nanotechnology-driven therapeutic platforms, such as engineered nanocarriers (e.g., liposomes, dendrimers, and polymeric micelles), enable precise drug targeting, reducing systemic toxicity and enhancing treatment efficacy [111]. While these innovations hold immense promise, the challenges remain in terms of scalability, regulatory approval, and long-term biocompatibility. Addressing these hurdles will be critical to translating these next-generation materials into clinical practice.

**Figure 5:**
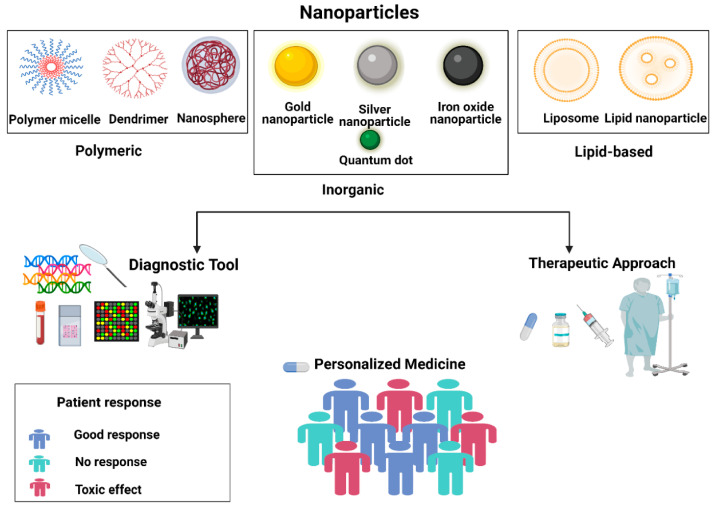
Schematic illustration of nanotechnology applications in personalized medicine. Copyright permission from reference no [112]. CC BY 4.0. https://doi.org/10.3390/jpm12050673.

Figure 5 provides a schematic representation of how nanotechnology is revolutionizing personalized medicine by enabling highly precise, patient-specific therapeutic and diagnostic approaches [112]. Nanotechnology-based systems, such as nanocarriers, nanosensors, and targeted drug delivery platforms, offer unparalleled advantages in optimizing treatment efficacy while minimizing adverse effects. Nanoparticles (e.g., liposomes, dendrimers, and polymeric micelles) can be engineered to deliver drugs directly to diseased tissues, enhancing bioavailability and reducing systemic toxicity. Moreover, nano-biosensors facilitate early disease detection by recognizing specific biomarkers at ultra-low concentrations, improving diagnostic accuracy and allowing timely interventions.

### 7.2. Integration with Personalized Medicine

The integration of micro- and nano-architectures into personalized medicine is poised to revolutionize the way healthcare is delivered. Personalized medicine focuses on tailoring medical treatment to the individual characteristics of each patient, such as their genetic makeup, lifestyle, and environmental factors. One of the most promising applications of micro- and nano-architectures in this context is the development of personalized drug delivery systems. Traditional drug therapies often have limited efficacy due to variability in how patients metabolize and respond to medications. Nanocarriers can be engineered to deliver drugs precisely to the right location, at the right time, and in the right dosage, based on the patient’s specific needs. By incorporating biomarker profiling, nanomedicines can be customized to target specific receptors or genetic mutations, enhancing therapeutic outcomes while minimizing side effects [113]. Moreover, nano-based diagnostic tools are enabling a more accurate and early detection of diseases, such as cancer, at the molecular level. These tools use nanoparticles to bind to specific biomarkers, providing a much higher sensitivity and specificity compared to traditional diagnostic methods. By combining nanodiagnostics with personalized treatment plans, physicians can identify disease risks early on and intervene before the disease progresses, allowing for precision medicine strategies that are proactive, rather than reactive [114]. In genetic therapies, micro- and nano-architectures play a critical role in facilitating the delivery of gene editing tools, such as CRISPR-Cas9. These materials ensure that the gene-editing tools can reach the specific cells that need to be modified, enhancing the efficiency and precision of genetic therapies. This integration allows for more effective treatments for genetic disorders, enabling personalized approaches to conditions that were once thought untreatable [115]. Additionally, micro- and nano-architectures are being used in cell-based therapies by enhancing the viability and function of transplanted cells, improving regenerative medicine outcomes.

### 7.3. Roadmap for Translational Research

The roadmap for translational research in the field of micro- and nano-architectures involves several key steps aimed at overcoming the current barriers to clinical application. The first step is to optimize fabrication techniques for scalability and reproducibility. While advancements in 3D printing, self-assembly, and lithographic techniques have made significant progress, the ability to produce these materials at a commercial scale without compromising on quality remains a challenge [116]. Researchers are working to streamline these fabrication methods and reduce the cost of production to make nano-engineered materials more accessible and economically feasible for widespread use. Next, improving biocompatibility and long-term stability of these materials is essential for their successful integration into clinical settings. While significant strides have been made in designing biocompatible materials, many still exhibit issues, such as toxicity, immunogenicity, or degradation over time. The development of self-healing materials and smart coatings that can adjust to the biological environment holds promise for addressing these challenges. Ensuring that these materials remain functional for long periods within the body, without causing harm, is a critical research focus moving forward. Finally, the regulatory framework needs to be adapted to accommodate the unique characteristics of micro- and nano-architectures. Current regulatory guidelines were not designed with these advanced materials in mind, and their novelty presents challenges for safety and efficacy testing. Researchers and regulatory agencies must collaborate to develop new standardized testing protocols for evaluating the biological interactions of nanomaterials and their clinical effectiveness. Furthermore, ethical concerns surrounding the use of nanomaterials, especially in gene editing and nano-based therapies, must be addressed to ensure that these technologies are used responsibly and safely. Ensuring public trust in these technologies will be key to their adoption and success. In conclusion, the future of micro- and nano-architectures in biomedical engineering looks highly promising, with continuous innovations driving the field forward. By addressing the current challenges and following a clear roadmap for the translational research, these technologies have the potential to revolutionize personalized medicine, diagnostics, and therapeutics, bringing us closer to the realization of truly personalized and precision-based healthcare solutions.

**Figure 6:**
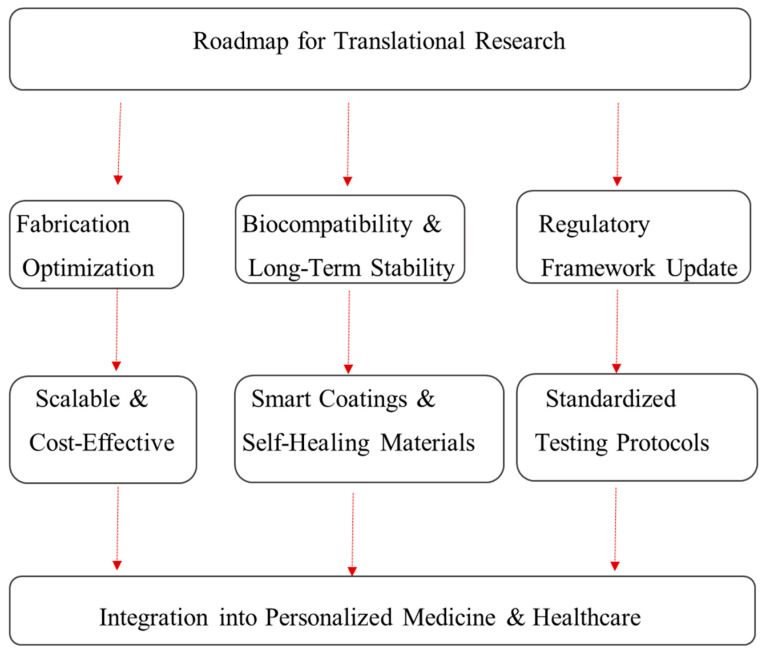
Translational roadmap for micro- and nano-architectures in biomedical engineering.

Figure 6 is the roadmap for the translational research on micro- and nano-architectures following a systematic approach to overcoming the existing challenges in biomedical applications. The first critical step involves optimizing fabrication techniques to ensure scalability and cost-effective production while maintaining precision and reproducibility. Advanced techniques, such as 3D printing, lithography, and self-assembly, must be refined to meet industrial-scale requirements. Next, biocompatibility and long-term stability play a pivotal role in clinical success, requiring the integration of self-healing materials and smart coatings to enhance durability and minimize immune responses. Furthermore, regulatory framework adaptation is essential, as the current safety and efficacy evaluation protocols are not fully equipped to handle the complexities of nanoscale architectures. Developing standardized testing methodologies will be crucial for gaining regulatory approval and ensuring public trust in these innovations. Ultimately, by addressing these core challenges, micro- and nano-architectures can transition from laboratory research to personalized medicine, diagnostics, and therapeutics, revolutionizing the future of healthcare with precision-based solutions.

## 8. Conclusions

### 8.1. Summary of Key Insights

1. Advancements in Fabrication: Techniques such as lithography, 3D printing, and self-assembly enable precise control over micro- and nano-architectures, enhancing material functionalities.
2. Therapeutic Applications: Engineered micro- and nano-architectures facilitate targeted drug delivery, regenerative medicine, and therapeutic implants, improving treatment efficiency and reducing side effects.
3. Diagnostic Innovations: Integration into biosensors, lab-on-a-chip devices, and imaging agents enhances disease detection with improved sensitivity, specificity, and real-time monitoring.
4. Key Design Parameters: Biocompatibility, mechanical resilience, and scalability are crucial for clinical translation and long-term stability of these materials.
5. Regulatory and Ethical Considerations: Progress is being made in standardizing the guidelines for safety, efficacy, and ethical transparency in the application of micro- and nano-architectures.
6. Future Prospects: The integration of these materials into personalized and precision medicine holds immense potential for transforming healthcare through tailored treatments and cost-effective solutions.

### 8.2. Final Remarks on Potential Impact

The continued development of micro- and nano-architectures is expected to profoundly impact various aspects of healthcare, especially in personalized medicine, precision diagnostics, and therapeutic strategies. As these materials evolve, they promise to offer solutions to some of the most pressing challenges in modern medicine, including targeting disease at the molecular level, improving therapeutic efficacy, and enhancing diagnostic accuracy. The integration of nanotechnology into biomedical devices and systems is leading to the next generation of smart medical devices capable of real-time monitoring and intervention, paving the way for more proactive and precise healthcare. The potential for bioprinting and gene editing in combination with nano-engineering also represents a breakthrough in organ regeneration, tissue repair, and gene therapies, promising to treat previously incurable diseases and injuries. However, for these technologies to realize their full potential, further efforts are needed to address scalability issues, ensure long-term stability, and overcome regulatory hurdles. Additionally, while the ethical and regulatory challenges are significant, they also present an opportunity to establish new standards and frameworks that ensure the safe and responsible application of these technologies. Ultimate, micro- and nano-architectures hold great promise for revolutionizing the future of medicine. By advancing the fundamental technologies, improving clinical translation, and addressing challenges in biocompatibility and scalability, these innovations will pave the way for the next generation of medical treatments, bringing us closer to a future of truly personalized healthcare and enhanced patient outcomes.

## Figures and Tables

**Table 1:** Summary of materials used in micro- and nano-architectures for biomedical applications.

Material Type	Examples	Key Properties	Applications in Biomedical Engineering	Ref.
**Polymeric Materials**	PLA, PCL, PEG, Chitosan, Gelatin	Biocompatibility, biodegradability, tunable mechanical properties	Drug delivery, tissue scaffolding, wound healing, controlled release systems	[7]
**Inorganic Materials**	Gold nanoparticles, Iron oxide, Silica	Stability, optical and magnetic properties	Imaging agents, contrast-enhancement, drug delivery, diagnostics	[8]
**Natural Biomaterials**	Collagen, Alginate, Silk fibroin	Biodegradable, bioactive, cell-interactive	Tissue engineering, wound healing, cell culture scaffolds	[9]
**Robotic/Bioinspired**	Self-assembled structures, 3D-printed scaffolds	High-precision, complex geometries	Fabrication of complex tissue architectures, personalized implants	[10]
**Nanostructures**	Carbon nanotubes, Quantum dots	High surface area; electrical, optical, and magnetic properties	Biosensing, diagnostics, targeted therapy, drug delivery	[11]

**Table 2:** Comparative overview of fabrication techniques for micro- and nano-architectures.

Fabrication Technique	Principle	Materials Used	Resolution	Advantages	Limitations	Applications
**Lithography-Based Methods**	Patterning of materials using light or electron beams to create nanoscale structures	Metals, polymers, semiconductors	Sub-micron to nanometer scale	High precision, high throughput	Expensive, limited to flat surfaces, complex setup	Fabrication of neural scaffolds, microfluidic platforms for organ-on-a-chip systems, nanopatterned surfaces for stem cell differentiation
**Photolithography**	Uses light exposure on a photosensitive material to form patterns	Polymers, silicon	~100 nm	Mature technology, high throughput	Requires expensive equipment, limited resolution	Microfabrication of lab-on-a-chip devices, biosensor arrays, and microelectrode arrays for neural interfaces
**Electron Beam Lithography (e-beam)**	Uses focused electron beam to create patterns directly on a substrate	Polymers, metals, semiconductors	Sub-10 nm	High resolution, direct patterning	Slow, high cost, complex equipment	Nanostructured substrates for biomolecule detection, nanopatterned surfaces for tissue engineering, fabrication of nanostructured drug carriers
**3D Printing Technologies**	Layer-by-layer additive manufacturing of materials to create complex 3D structures	Polymers, ceramics, hydrogels, metals	Micro- to millimeter scale	Flexible, customizable, cost-effective for prototyping	Limited resolution, material limitations, slow process	Patient-specific implants, 3D-printed vascularized tissue constructs, bioactive scaffolds for tissue regeneration
**Stereolithography (SLA)**	Uses UV light to cure liquid resin into solid layers	Photopolymer resins	~50–100 µm	High resolution, fast prototyping	Limited material choice, post-processing required	Dental implants, surgical guides, patient-specific bone grafts
**Two-Photon Polymerization**	Uses focused laser to polymerize materials in a highly localized manner	Photopolymer resins	<100 nm	High resolution, 3D printing of complex structures	Slow, limited material options, high cost	Fabrication of microvascular networks, nanostructured scaffolds for neural regeneration
**Self-Assembly Techniques**	Spontaneous organization of molecules or particles into desired structures	Nanoparticles, block copolymers, biomolecules	Nanometer scale	Low cost, minimal energy input, scalable	Requires precise control over conditions, limited scalability	Smart drug carriers, bioinspired membranes for controlled drug release, self-assembled peptide hydrogels for wound healing
**Molecular Self-Assembly**	Molecules spontaneously form ordered structures due to intermolecular interactions	Organic molecules, nanoparticles	Nanometer scale	Simple, energy-efficient, cost-effective	Limited control over large-scale organization, slow process	Nanoparticle synthesis for targeted therapy, biomimetic hydrogels for wound healing
**Block Copolymer Self-Assembly**	Block copolymers self-assemble into nanoscale structures based on phase separation	Block copolymers, polymers	~10–100 nm	High precision, versatile	Requires specific conditions, material limitations	Nanostructured drug carriers, porous scaffolds for regenerative medicine, biomimetic membranes for biosensing

**Table 3:** Comparative analysis of stimuli-responsive materials: mechanisms, applications, advantages, and limitations.

Type of Stimuli-Responsive Material	Stimulus	Mechanism	Applications	Advantages	Limitations	Ref.
**pH-Responsive Polymers**	pH changes (e.g., acidic or alkaline environments)	Protonation or deprotonation of functional groups (e.g., carboxyl or amine groups) changes material solubility or swelling behavior.	Tumor-targeted drug delivery, gastrointestinal DDS.	High specificity in acidic environments (e.g., tumors)	Limited to environments with significant pH gradients; risk of premature degradation.	[71]
**Thermo-Responsive Polymers**	Temperature variations	Phase transition occurs at critical solution temperature (LCST or UCST), altering solubility.	Injectable hydrogels for tissue regeneration, smart drug carriers.	Minimally invasive; temperature-sensitive control	Potential loss of function in fluctuating body temperature conditions.	[72]
**Light-Responsive Materials**	UV, visible, or NIR light	Photoisomerization or photothermal conversion induces structural changes or triggers release.	Photothermal therapy, on-demand drug release, bio-imaging.	High spatiotemporal control; non-invasive activation	Limited tissue penetration depth for light (especially UV or visible); phototoxicity.	[73]
**Magneto-Responsive Materials**	Magnetic fields	Magnetic nanoparticles (e.g., Fe_3_O_4_) heat under an alternating magnetic field or align for targeted movement.	Hyperthermia therapy, guided drug delivery.	Remote activation; deeper penetration possible	Requires external magnetic fields and specialized equipment.	[74]
**Electro-Responsive Polymers**	Electric fields	Change in electrical potential alters molecular alignment or triggers ion transport.	Neural tissue engineering, electroactive drug release.	Precise electrical control; compatibility with bioelectronics	Risk of local heating or cell damage from high-intensity electrical fields.	[75]
**Mechanical-Responsive Polymers**	Pressure, shear, or strain	Changes in structure (e.g., micropores open/close under stress) or release of encapsulated drugs.	Wound healing dressings, wearable sensors.	Responds to external forces; no additional stimuli needed	Difficult to achieve controlled and uniform response under variable mechanical forces.	[76]
**Enzyme-Responsive Polymers**	Specific enzymes	Enzymatic degradation of polymer matrix or release of drugs upon enzyme-triggered cleavage.	Cancer therapy (elevated enzyme levels in tumors), infection-responsive systems.	High specificity to biological microenvironments	Limited by enzyme activity and concentration in target tissue.	[77]
**Multi-Responsive Materials**	Combination of stimuli	Integration of dual or multiple responses (e.g., pH + light, temperature + magnetic fields).	Cancer therapy, smart scaffolds, controlled release.	Synergistic response; greater versatility	Complex design and fabrication; difficulty in balancing stimuli sensitivity.	[78]

**Table 4:** Comparative analysis of diagnostic applications: biosensing, imaging, and microfluidic technologies.

Application	Key Technology	Mechanism	Advantages	Limitations	Ref.
**Biosensing and Detection Technologies**	**Nano-Biosensors**	Detection of disease biomarkers (e.g., proteins, DNA, RNA) using nanoparticles or nanomaterials (e.g., gold, graphene) to enhance sensitivity.	High sensitivity, rapid detection, ability to detect low concentrations of biomarkers.	Sensitivity can be affected by non-specific binding; need for precise functionalization of nanoparticles to improve selectivity.	[93]
	**Integration with Point-of-Care Devices**	Portable devices that integrate nano-biosensors for onsite disease detection (e.g., glucose testing, cancer biomarker detection) in real-time.	Enables rapid diagnostics in resource-limited settings; portable and easy to use.	Limited in terms of detectable diseases due to sensor specificity, sample preparation challenges, and biomarker stability; difficulties in detecting multiplexed biomarkers in complex biological samples.	[94]
**Imaging and Contrast-Enhancing Agents**	**Nanoparticle-Based Contrast Agents**	Use of nanoparticles (e.g., gold, silica, iron oxide) to enhance imaging in techniques such as MRI, CT, and ultrasound.	Enhanced imaging quality, improved tissue contrast, targeted imaging for early detection.	Risk of nanoparticle toxicity; challenges in controlling biodistribution and clearance from the body.	[95]
	**Quantum Dots and Fluorescent Nanoparticles**	Fluorescent nanoparticles that provide high-resolution imaging with multi-color capability.	Superior resolution, multiplexing capabilities, non-invasive monitoring.	Potential toxicity in vivo, photobleaching over time affecting long-term imaging accuracy.	[96]
**Lab-on-a-Chip and Microfluidic Platforms**	**Microfluidic Devices**	Miniaturized systems that use small-scale fluid handling (microchannels) to analyze biological samples with high efficiency.	High throughput, low sample and reagent consumption, integration with other diagnostic tools.	Complexity in device design; limitations in large-scale manufacturing and high initial costs.	[97]
	**Lab-on-a-Chip (LOC) Technology**	Integration of various laboratory functions (e.g., PCR, immunoassays) on a single chip for rapid diagnostics.	Faster diagnostics, portable, and requires minimal sample handling.	High manufacturing cost, technical challenges in integrating multiple functions onto a single chip.	[98]

**Table 5:** Key design parameters, materials, advantages, and challenges of micro- and nano-architectures.

Design Parameter	Key Considerations	Materials/Technologies	Advantages	Challenges/Limitations	Ref.
**Biocompatibility and Safety**	Interaction with biological systems without causing toxicity or inflammation.	Gold nanoparticles, graphene, biocompatible polymers (e.g., PEG), hydrogels, carbon nanotubes.	Reduced immune response, safe degradation products, compatibility with tissue.	Risk of cytotoxicity, potential immune activation from carbon nanotubes, long-term stability issues, toxicity of degradation products.	[103]
	Surface modification to enhance compatibility and reduce immunogenicity.	Surface-functionalized nanoparticles, biodegradable polymers.	Enhances material stability, prevents immune activation, improves circulation.	Requires sophisticated surface engineering, in vivo validation needed, risk of altered bio-distribution.	[104]
**Mechanical Properties and Resilience**	Strength, elasticity, and durability under physiological conditions.	Elastomers, hydrogels, bioactive ceramics, carbon nanotubes, graphene oxide.	High mechanical performance, mimics biological tissue characteristics.	Difficulty in matching mechanical properties with native tissues; mechanical fatigue in long-term applications.	[105]
	Self-healing capability for longevity and functionality.	Self-healing polymers, dynamic hydrogels, supramolecular hydrogels.	Enhanced durability, recovery from mechanical damage, increased lifespan.	Complexity in designing self-healing materials, limited scalability, potential changes in mechanical strength over time.	[106]
**Scalability and Cost-Effectiveness**	Ability to produce materials at scale without sacrificing quality.	3D printing, roll-to-roll processing, photolithography, self-assembly.	Potential for large-scale, low-cost manufacturing; high throughput.	Limited scalability of some techniques (e.g., photolithography), expensive fabrication steps, batch-to-batch variations.	[107]
	Cost considerations for large-scale production.	Carbon-based materials, biodegradable polymers, low-cost metals.	Cost-effective materials and manufacturing methods, easily available.	High material costs (e.g., gold nanoparticles), complex processing for certain biodegradable polymers.	[108]
	Ensuring reproducibility across large batches.	Mass production techniques, automated systems (e.g., inkjet printing, microcontact printing).	Consistent quality across large-scale production.	Variability in material properties across different batches, challenges in high-precision manufacturing, need for rigorous quality control.	[109]
